# Gender and Age Moderate the Effects of Partner Substance Use on Problem Drinking in Adulthood

**DOI:** 10.1093/geroni/igaa057.1294

**Published:** 2020-12-16

**Authors:** Sara Miller, David Almeida, Jennifer Maggs

**Affiliations:** Pennsylvania State University, University Park, Pennsylvania, United States

## Abstract

The current study examined whether partner substance use problems predict problem drinking and how gender and age moderate this relationship. Problem drinking refers to alcohol use resulting in alcohol dependence or health and social consequences. Participants were adults (n=2142, 53% female, mean age=54, range= 33-83) from Wave 2 of the Midlife Development in the United States (MIDUS) Study. Participants reported on both past 12-month problem drinking (e.g., emotional problems from drinking, urges to drink, month or “much time” drinking, drinking more to get effects, drinking more than intended, and alcohol-related role interference) and partner substance use problems. Results indicated that 22.2% of the sample reported at least one problem drinking behavior in the past year. Multiple linear regression analysis revealed a significant interaction between gender and partner substance use problems (b=0.05, p=0.01) such that for males having a partner with substance use problems was a risk factor for their own problem drinking. However, a three-way interaction with gender, age, and partner substance use problems (b=-0.41, p<0.01) indicated that partner substance use problems might have both gender and age-specific effects on problem drinking. Exploratory analyses of this interaction indicated that with age partner substance use problems might no longer promote risk for male problem drinking. Older adults are especially sensitive to the effects of alcohol for reasons such as lower tolerance, medication interaction, and health conditions. There is thus a need for identifying age-relevant factors associated with these drinking behaviors for intervention and prevention efforts.

